# The use of peptides for immunodiagnosis of human Chagas disease

**DOI:** 10.1007/s00726-024-03394-6

**Published:** 2024-05-02

**Authors:** Anna Julia Ribeiro, Kamila Alves Silva, Lucas da Silva Lopes, Carlos Ananias Aparecido Resende, Carolina Alves Petit Couto, Isadora Braga Gandra, Isabela Amorim Gonçalves Pereira, Isabelle Caroline Dos Santos Barcelos, Sabrina Paula Pereira, Sandra Rodrigues Xavier, Grasiele de Sousa Viera Tavares, Juliana Martins Machado, Mariana Campos Da Paz, Miguel Angel Chávez-Fumagalli, Eduardo Antonio Ferraz Coelho, Rodolfo Cordeiro Giunchetti, Ana Thereza Chaves, Walderez Ornelas Dutra, Ana Alice Maia Gonçalves, Alexsandro Sobreira Galdino

**Affiliations:** 1 Laboratório de Biotecnologia de Microrganismos, Universidade Federal de São João Del-Rei, Divinópolis, e Instituto Nacional de Ciência e Tecnologia em Biotecnologia Industrial (INCT-BI), Divinópolis, MG 35501-296 Brazil; 2 Programa de Pós-Graduação em Ciências da Saúde: Infectologia e Medicina Tropical, Faculdade de Medicina, Universidade Federal de Minas Gerais, Belo Horizonte, MG 30130-100 Brazil; 3 Laboratório de Bioativos e Nanobiotecnologia, Universidade Federal de São João Del-Rei, Divinópolis, MG 35501-296 Brazil; 4 Computational Biology and Chemistry Research Group, Vicerrectorado de Investigación, Universidad Católica de Santa María, Arequipa, 04000 Peru; 5 Laboratório de Biologia das Interações Celulares, Departamento de Morfologia, Instituto de Ciências Biológicas, Universidade Federal de Minas Gerais, e Instituto Nacional de Ciência e Tecnologia em Doenças Tropicais (INCT-DT), Belo Horizonte, MG 31270-901 Brazil

**Keywords:** Chagas disease, *Trypanosoma cruzi*, Serodiagnosis, Imunodiagnosis; peptide

## Abstract

Chagas disease, caused by the protozoa *Trypanosoma cruzi*, continues to be a serious public health problem in Latin America, worsened by the limitations in its detection. Given the importance of developing new diagnostic methods for this disease, the present review aimed to verify the number of publications dedicated to research on peptides that demonstrate their usefulness in serodiagnosis. To this end, a bibliographic survey was conducted on the PubMed platform using the keyword “peptide” or “epitope” combined with “Chagas disease” or “*Trypanosoma cruzi*”; “diagno*” or “serodiagnosis” or “immunodiagnosis”, without period restriction. An increasing number of publications on studies employing peptides in ELISA and rapid tests assays was verified, which confirms the expansion of research in this field. It is possible to observe that many of the peptides tested so far originate from proteins widely used in the diagnosis of Chagas, and many of them are part of commercial tests developed. In this sense, as expected, promising results were obtained for several peptides when tested in ELISA, as many of them exhibited sensitivity and specificity values above 90%. Furthermore, some peptides have been tested in several studies, confirming their diagnostic potential. Despite the promising results observed, it is possible to emphasize the need for extensive testing of peptides, using different serological panels, in order to confirm their potential. The importance of producing an effective assay capable of detecting the clinical stages of the disease, as well as new immunogenic antigens that enable new serological diagnostic tools for Chagas disease, is evident.

## Introduction

Chagas disease (CD), a neglected tropical disease mainly associated with poverty, is caused by the hemoflagellate protozoan *Trypanosoma cruzi* (Chagas 1909). Parasite transmission can occur through several forms, including vectorial, congenital, oral, blood transfusion, and organ transplants (Bern et al. 2019). Of the 21 countries where CD is endemic, 17 managed to interrupt vector transmission in households throughout the countries, or in part of their national territories (PAHO/WHO 2023). However, effective monitoring of protozoan transmission is complex due to the zoonotic *T. cruzi* cycle (Shikanai-Yasuda and Carvalho 2012). The disease can cause severe visceral damage, involving damage to the heart (Nunes et al. 2018) and nervous system, as well as in the gastrointestinal tract (De Salazar et al. 2022; Forsyth et al. 2021; Rassi et al. 2010).

Although most cases are concentrated in Latin America, there is a notable increase in cases in non-endemic countries in North America, Europe and the Western Pacific regions, making CD a global public health problem, with great social impact (Lidani et al. 2019; Gómez-Ochoa et al. 2022). Some circumstances involved in the expansion of CD cases are related to the intensification of migratory movements, often motivated by social inequalities (Avaria et al. 2022; Marin-neto et al. 2023). Furthermore, changes in climatic and environmental factors favored the transmission of the parasite, as this directly influences its life cycle and geographic distribution (Medone et al., 2015). Furthermore, CD represents a major threat to public health worldwide as there are no adequate drugs for treatment or vaccines. Currently, the global amount spent on infected individuals is estimated at US$ 24 to 73 billion, with an annual value of US$ 4,660/person (Ferreira et al. 2022; Mucci et al. 2017; Lee et al. 2013). Despite these expenses, the population still faces many challenges regarding access to the healthcare system and the lack of subsidies to cope with the disease (Suárez et al. 2022). Currently, the disease affects 6 to 8 million people and causes 10,000 deaths per year, primarily in Latin American countries (WHO 2023). Among the countries reporting CD cases, Argentina, Bolivia, Brazil, Colombia, and Mexico are the ones with the highest prevalence (Medeiros et al. 2022). In Brazil, which has the third highest incidence rate in Latin America, it is estimated that there are approximately one million people infected with *T. cruzi*, representing one of the four main causes of death from infectious and parasitic diseases in the country (Brasil 2022).

The knowledge about *T*. *cruzi* life cycle and disease pathogenesis allowed the disease to be divided into an acute and a chronic phase (Suárez et al. 2022; Coura 2013; Segovia et al. 1916; Chagas 1909). The acute phase is characterized by a high parasitemia, occurrence and nonspecific symptoms where it is also possible to visualize parasites in the peripheral blood. The most appropriate methods for diagnosing the disease at this phase are direct and indirect parasitological tests, accompanied by serological tests (PAHO/WHO 2019). At this stage, diagnostic methods are still very time-consuming, demonstrating the importance of developing a differential serological test (SESPA 2023). However, indirect tests applied in the acute phase have low sensitivity and depend entirely on professional training, ending up not being a practical strategy. Furthermore, most cases go unnoticed due to the lack of specific symptoms and the fact that many individuals have little access to information (Schijman et al. 2022). Importantly, if CD can be identified early, the individual has a better chance of having good therapeutic responses and perhaps a satisfactory clinical evolution before the protozoan triggers irreversible complications and eventually becomes fatal (PAHO/WHO 2023).

The chronic phase can be symptomatic or asymptomatic, which will determine the strategies and diagnostic tests to be used (Schijman et al. 2022). Chronic individuals may present cardiovascular and/or gastrointestinal tract, and nervous system changes at different stages, which are the leading causes of death from the disease (Forsyth et al. 2021; Ledezma et al. 2020). The level of parasitemia in this phase decreases considerably, rendering the use of microscopic diagnosis an unreliable diagnostic method (Suárez et al. 2022). Serological tests are more often used as a diagnostic tool to achieve greater sensitivity and specificity (Balouz et al. 2017). Three conventional types of serological tests are approved by the WHO: (*i*) the enzyme-linked immunosorbent assay (ELISA); (*ii*) indirect immunofluorescence assay (IIF); and (*iii*) indirect hemagglutination assay (IHA) (Abras et al. 2022; PAHO/WHO 2019). The effectiveness of these available tests may vary, especially due to the different antigens applied and diagnostic confirmation for the chronic phase, following the guidelines of the Pan American Health Organization and the Centers for Disease Control (CDC), requires a positive result in at least two different tests and, in case of disagreement, a third test must be incorporated (PAHO/WHO 2019). Although serological tests are considered the gold standard for the diagnosis of chronic CD (PAHO/WHO 2019), the need to confirm the disease by at least two tests involves great effort in manufacturing reagents and is, therefore, expensive (Forsyth et al. 2021). It is of fundamental importance to incorporate an adequate diagnosis with high sensitivity and specificity to assist in surveillance actions aimed at preventing new cases (Santos et al. 2020).

Determining new biological markers that can provide preliminary recognition, as well as disease screening, is, thus, very useful (Mucci et al. 2017). The use of peptides has been reported in many studies as a resource for CD diagnosis, in addition to diagnosing other infectious diseases (Castiglione et al. 2022; Falconi-Agapito et al. 2022; Serena et al. 2022; Lorenzo et al. 2021; Li et al. 2020). Peptides consist of short portions of amino acids that have signaling capacity and essential biomolecules for various biological processes with high biomolecular recognition and high binding affinity for a wide range of specific targets (Apostolopoulos et al. 2021). Due to the potential of using peptides in CD diagnosis, in addition to the great interest of the diagnostic industry in the development and application of peptides in diagnostic kits, the focus of this review is a discussion on the studies that used peptides applied in ELISA assays and rapid tests as a tool for CD diagnosis.

## Methodology

The search for was carried out using the PubMed database, including all papers published to April/2024. The descriptors used were: ((trypanosoma cruzi [Title/Abstract]) AND (imunodiagnosis[Title/Abstract])) AND (peptide[Title/Abstract]); ((trypanosoma cruzi [Title/Abstract]) AND (serodiagnosis[Title/Abstract])) AND (peptide[Title/Abstract]); ((trypanosoma cruzi[Title/Abstract]) AND (diagno*[Title/Abstract])) AND (peptide[Title/Abstract]); ((chagas disease [Title/Abstract]) AND (diagno*[Title/Abstract])) AND (peptide[Title/Abstract]); ((chagas disease [Title/Abstract]) AND (imunodiagnosis[Title/Abstract])) AND (peptide[Title/Abstract]); and ((chagas disease [Title/Abstract]) AND (serodiagnosis[Title/Abstract])) AND (peptide[Title/Abstract]). The selected articles were screened using inclusion and exclusion criteria, reviewed by two different readers. Animal articles, bibliographical reviews, case studies, epidemiological reviews, molecular and serological diagnoses of other diseases, editorials, duplicate articles, and articles related to other subjects were excluded. Only those articles employing ELISA or rapid test assays using peptides for CD diagnosis were included, regardless of whether there was a comparison with commercial tests or whether there was more than one test.

## Peptides and their advantages as a diagnostic tool

Amino acids and peptides were mentioned for the first time in the nineteenth century (Vickery and Schmidt 1931; Hansen 2015). As a result, German chemists Hermann Emil Fischer and Franz Hofmeister developed an important study where they introduced several concepts about peptides and polypeptides (Fourneau and Fischer 1901; Wieland and Bodanszky 1991). Currently, peptides have applications in various health areas, such as diagnosis, vaccines, and therapy (Liu et al. 2022; Al-Azzam et al. 2020; Fisher et al. 2019; Link et al. 2017), and can be obtained through chemical synthesis and enzymatic hydrolysis (Akbarian et al. 2022). In addition, the use of peptides requires prior investigation that needs a global genomic and proteomic analysis of organisms (Pandey et al. 2021; Al-Azzam et al. 2020). To this end, different tactics have been developed to identify specific amino acid sequences within molecules of interest, including the phage display methodology (Zhang et al. 2022; Lechner et al. 2019; Piggott and Karuso 2016; Rangel et al. 2012) and bioinformatics analyses (Pandey et al. 2021).

Among the various applications, the use of peptides has shown great potential for disease diagnosis due to their sensitivity and specificity for different targets (Al-Azzam et al. 2020), offering more advantages when compared to the native antigen (Joshi et al. 2013; Saravanan et al. 2004). The use of peptides is important in clinical diagnosis because they are difficult to undergo variations, and can be easily manipulated, stored, and produced on a large scale. Peptides can be applied in several detection methods, such as lateral flow devices, and microarray or immunoenzymatic assay (ELISA), the latter being the most adopted diagnostic tool (Pandey et al. 2021). The post-pandemic effects of COVID-19, had repercussions on the need to develop new technologies for the mass production of effective diagnostic tools (Safiabadi Tali et al. 2021). Therefore, different peptides were tested and shown to be reactive with the serum of infected individuals (Cortés-Sarabia et al. 2022), reinforcing the importance of these molecules in the field of diagnostics. Furthermore, the use of peptides in immunodiagnosis has already shown promising results in numerous infectious diseases, such as strongyloidiasis, infectious bronchitis, blue tongue, and AIDS (Jackwood and Hilt 1995; Gonzalez et al. 1997; Feliciano et al. 2014; Saxena et al. 2012).

In fact, it is expected that the global peptide-based diagnostics market will grow by 9.6% from 2022 to 2027, with predictions of reaching a value of USD 11.4 billion (GME 2023). The use of peptides has been increasingly proposed to replace the customary methods that use recombinant proteins (Pandey et al. 2021). With this in mind, short peptides, containing more than eight amino acids, have several advantages when used in the detection of specific antibodies, as they facilitate synthesis, biodegradability, and biocompatibility, in addition to offering stability and economy, considering manufacturing costs are greater depending on the size of the amino acid sequence (Apostolopoulos et al. 2021; Brown et al. 1997). Therefore, use of peptides has proven to be very advantageous in detecting diseases and can significantly contribute to the management of healthcare systems and patient monitoring.

## Peptide-based ELISA assays

ELISA was developed in 1971 by scientists Eva Engvall and Peter Perlmann (Engvall and Perlmann 1971), with the intention of validating the presence of molecules through antigen–antibody interaction. This discovery led to the technique being improved and gave rise to different types of ELISA that are now widely used in peptide and protein research, given that it is an extremely effective test (Aydin 2015; Engvall 2010). This method has been widely used in the investigation of new techniques to diagnose CD and with good results. At the moment, there are 20 studies in the literature using the peptide technique as a probable serological marker capable of replacing traditional methods, and the following topics summarize the results of each one.

A study published by Burns et al. (1992) was the first to use a peptide for CD diagnosis. The authors reported a repetitive peptide within an immunodominant *T. cruzi* protein. This peptide was obtained through chemical synthesis and its reactivity with positive serum samples was tested in an ELISA assay. For this purpose, 129 positive serum samples were used, in addition to 32 serum samples from healthy individuals as negative control. Results showed that synthetic TcD was recognized by 96.7% of the CD serum samples. Furthermore, only 3.3% of sera from non-Chagas individuals were reactive with the peptide. In addition, synthetic TcD was not identified by any sera from individuals with leishmaniasis. Moreover, the performance of the synthetic peptide was compared with that of a recombinant protein, where the synthetic peptide showed a comparable capacity to be recognized by positive serum samples, as well as displaying improved specificity.

Subsequently, Peralta et al. (1994) tested the mixture of two synthetic peptides from *T. cruzi*, called TcD, previously tested by Burns et al. (1992), and PEP2, obtained through chemical synthesis. The peptides were analyzed using a panel with 179 samples from infected individuals residing in a CD endemic area, as well as 81 serum samples from healthy individuals used as a negative control. Serum samples from individuals with other infectious diseases that might show cross-reaction with *T. cruzi* antigens were also used. When peptides were tested individually, the TcD and PEP2 sensitivity values were 93% and 91%, respectively. A mixture of both was evaluated and a sensitivity greater than 99% was observed. They also evaluated commercial tests IHA and IFA, using crude extract of *T. cruzi*, and obtained specificities of 99%, when using IHA, and 94%, when applying IFA.

Aznar et al. (1995) evaluated the diagnostic capacity of peptide R-13, corresponding to the C-terminal sequence of a *T. cruzi* ribosomal P protein, which was obtained through chemical synthesis. Serum from 161 Chagas individuals, 207 blood bank samples, and 20 serum samples from non-Chagas diseases were used to access peptide reactivity. Results indicated that 86% of congenital and 60% of chronic serum samples recognized R-13. Furthermore, all acute sera showed anti-*T. cruzi* IgM antibodies. However, R-13 was not recognized by serum samples from asymptomatic Chagas individuals or individuals with digestive symptoms. Moreover, 49% of serum samples from blood donors showed reactivity with R-13.

Later, Houghton et al. (1999) described the diagnostic use of two synthetic peptides, 2/D/E and TcLo1.2, which were obtained through chemical synthesis. To evaluate their reactivity, 240 positive samples from different geographic sources and 149 serum samples from healthy individuals were used. Results showed that the 2/D/E peptide demonstrated a sensitivity of 99.6% and a specificity of 9.33%. In relation to the TcLo1.2 peptide, a greater reactivity was observed with enhanced specificity for *T. cruzi*. A comparison was made between the specificities of the peptide and of *T. cruzi* lysate, where the TcLo1.2 peptide demonstrated a high degree of specificity.

Continuing the studies with 2/D/E-2 peptide, Betonico et al. (1999) evaluated its diagnostic capacity using 40 positive and 107 negative serum samples. Moreover, serum samples from non-Chagas individuals were also used to verify cross-reactions. The synthetic peptide was recognized by all serum samples from individuals with acute infection. However, when analyzing serum samples from individuals with chronic infection, only 12.9% of the serum samples reacted with the 2/D/E peptide. In addition, serum samples from healthy individuals and from non-Chagas diseases did not recognize the 2/D/E peptide. Furthermore, the synthetic peptide performance was compared to the diagnostic performance of the alkaline extract, in which the synthetic peptide showed reduced sensitivity with improved specificity.

Later, Gironès et al. (2001) tested the reactivity of two peptides, isolated from a previous study, and evaluated their potential as CD biomarkers. The peptides, called R3 and S1, were obtained by chemical synthesis and are derived from the dominant autoantigen (Cha) and an acute phase immunogenic antigen, respectively. Reactivity of the peptides was evaluated using a panel of 79 sera from infected individuals and 10 sera healthy individuals. Serum samples from unrelated illnesses were also used. The R3 peptide results revealed sensitivity of 92.4% and specificity of 100%. In relation to S1, the results obtained low recognition of antibody titers for this peptide. The reactivity was similar to other commercial serological tests that used *T. cruzi* extracts. It was also observed that anti-R3 antibody levels increased as the disease progressed and decreased significantly when individuals were on treatment.

Thomas et al. (2001) analyzed the immunological response of the KMP1 protein, during the CD infectious process. To better understand the regions involved in the recognition of this protein, seven peptides, designated 12,636, 12,637, 12,638, 12,639, 12,640, 12,641, and 12,642, were developed by chemical synthesis and their diagnostic performance was tested using 20 serum samples from infected individuals and 10 negative controls. The results indicated that only peptides 12,638 and 12,642 were able to recognize CD serum samples. However, sensitivity and specificity values were not provided.

In the study developed by Hernández-Marin et al. (2003), an evaluation of the reactivity of three synthetic peptides from *T. cruzi*. Peptides p17 (R-COOH), p17 (RCONH_2_) and p18, originated from antigenic regions of the parasite, were tested in ELISA and compared with a natural antigen. Then, peptides were tested using 20 positive serum samples and 20 serum samples from healthy individuals. Results showed that all peptides presented 100% of sensitivity. Furthermore, all peptides have 100% specificity when compared to natural antigens.

Afterwards, Hernández-Marin et al. (2006) tested the reactivity of synthetic peptides that can be used to identify antibodies in Chagas individuals. Two peptides, P1 and P2, each of which contained immunodominant repeat B cell epitopes from *T. cruzi*, were chemically synthesized. They were evaluated using 82 positive serum samples, including Colombian and Brazilian samples, and 44 Chagas-negative serum samples. The results indicated that P1 recognized 69% of the positive samples from Colombia and 86% of the positive samples from Brazil. Regarding the P2 results, 49% of the positive samples from Colombia and 89% of the positive samples from Brazil recognized P2.

Camussone et al. (2009) performed a rational selection of antigenic peptides with a diagnostic potential for CD. In their work, peptides designated RP1, RP2, and RP5 were tested alone or in combination. To access the peptides’ reactivity, 32 samples from infected individuals and 32 Chagas-negative serum samples were used. The results showed that all peptides were recognized by the positive serum samples. Moreover, peptides mixtures showed a greater discrimination limit as compared to the results when peptides were analyzed alone. However, the peptides’ performance was lower as compared to the diagnostic performance of multiepitope protein which were constructed using these same peptides.

Next, Thomas et al. (2012) tested the reactivity of five peptides exposed in a TcCA-2 antigen of *T. cruzi* in sera from symptomatic and asymptomatic Chagas-positive individuals. The peptides, designated 3972, 6303, 3973, 3963, and 6173, were synthesized using a simultaneous solid phase multiple peptide method. Their reactivity was analyzed through an ELISA using a panel of 97 positive sera and 30 negative sera. The results showed that peptides 3972, 6303, and 3973 demonstrated 90% sensitivity. Subsequently, they tested the IgG reactivity against only the 3973 peptide, which demonstrated a specificity greater than 98%.

Later, Longhi et al. (2012) evaluated the diagnostic capacity of different peptides, referred to as P013, R13, JL18, JL19, and P0b. Peptide R13 was derived from the C-terminal 13 amino acids of TcP2b, while P013 and P0b were derived from the C-terminal region of the TcP0 protein. Peptide JL18 and JL19 were derived from the *T. cruzi* recombinant JL9 protein. In their study, 228 positive serum samples and 108 serum from individuals without infection were used. Among the tested peptides, P013 showed high specificity. However, it also presented low sensitivity. Moreover, the peptides’ performance was generally lower as compared to the diagnostic performance of *T*. *cruzi* lysate and JL7 protein.

Mendes et al. (2013) performed a genomic screening with the aim of recognizing B cell epitopes and pointing out new serotyping targets. The immunoscreening of 150 high-scoring peptides resulted in the identification of 36 new epitopes and four peptides were chemically synthesized, referred to as C6_30_cons, A6_30_col, Peptide B2_30_y, and Peptide B9_30_cl. Afterward, the peptides were validated using serum samples from 10 Chagas individuals, 56 samples from individuals infected by untyped parasites, and 24 serum samples from healthy individuals. Among the tested peptides, A6_30_col showed the best diagnostic performance, with 100% sensitivity and 91.9% specificity. Peptide C6_30_cons also showed a promising capacity for detecting CD, with 95.8% sensitivity and 88.5% specificity.

Next, Bottino et al. (2013) performed an analysis to map the epitopes of proteins already characterized and known to be highly antigenic, namely the cytoplasmic repetitive antigen (CRA) and flagellar repetitive antigen (FRA). A library was created with serum from Chagas individuals, and based on the observed reactivity, three peptides were identified and synthesized in a solid phase, CRA-1, CRA-2, and FRA-1 epitopes. Thirty-one samples from infected individuals and 12 negative samples were used to evaluate the peptides’ diagnostic performance. Serum samples from leishmaniasis-infected individuals were also used to access cross-reactions. CRA-1 and CRA-2 peptides showed 100% sensitivity and specificity, while FRA-1 presented 91.6 sensitivity and 60% specificity.

Later, Bhattacharyya et al. (2014) made a comparison of the genetic diversity of *T. cruzi* to form synthetic peptides based on previously described *T. cruzi* TSSA lineage-specific amino acid sequences. Five peptides, designated TSSApep-I, TSSApep-II/V/VI, TSSApep-III, TSSApep-IV, andTSSApep-V/VI, were chemically synthesized. Serum samples from 186 infected individuals and 31 serum samples from healthy individuals were used. Among the tested peptides, TSSApep-II/V/VI demonstrated greater recognition by serological samples from individuals from different geographic regions.

Balouz et al. (2015) prepared a mapping to identify motifs within a *T*. *cruzi* surface antigen. The identified peptide, p36-50, was obtained through chemical synthesis. In order to access its diagnostic performance, 70 serum samples from infected individuals and 38 serum samples from healthy individuals were used. Although sensitivity and specificity values were not provided, the results showed that p36-50 was recognized by the positive samples, with a similar diagnostic performance as compared to a recombinant protein that was also tested in the study.

Next, Mucci et al. (2017) developed a proof-of-principle multiplex diagnostic kit using different validated peptides. More than 2,000 candidate peptides have been identified using a *T. cruzi*/Chagas HD peptide microarray. After analysis, 28 peptides were chemically synthesized and further tested in an ELISA assay. For this purpose, serum samples from 62 infected individuals were used, along with 16 serum samples from healthy individuals used as a negative control. Initial tests showed sensitivity and specificity values of all peptides, ranging from 3.23 to 91.98% and 93.8 to 100%, respectively. The best-performing peptides, designated pc1, pc2, pc3, p6, p7, p13, and p24, were used to form a new multiepitope recombinant protein, which showed improved sensitivity and specificity values.

Elisei et al. (2018) used bioinformatics analyses to select three B cell epitopes to be used as antigens for ELISA assays. These peptides, referred to as peptide 1, peptide 2, and peptide 3, were chemically synthesized and tested with 53 sera from Chagas-positive individuals. Additionally, 25 serum samples from healthy individuals were used as a negative control, along with 45 serum samples from individuals with leishmaniasis that were used to access cross-reactions. Among the tested antigens, peptide 2 showed the best diagnostic performance with 100% sensitivity and 97.14% specificity. Peptide 1 showed sensitivity and specificity values of 60.38% and 100%, respectively. Regarding peptide 3 results, a 72.86% of sensitivity was observed, with specitivity value determined as 67.14%. In addition, peptide 2 and peptide 3 were combined to form Mix II, which showed 100% sensitivity and specificity. When compared with laboratory tests IHA, IFA and ELISA, peptide 2 showed a greater diagnostic performance.

Ruiz-Marvéz et al. (2020) performed a B epitope prediction to investigate the possible linear epitopes of the Tc964 protein. After bioinformatics analyses, two peptides, TcNV and TcKP, were obtained using chemical synthesis and their reactivity was tested by ELISA using 63 serum samples from Chagas-positive individuals at different disease stages and six serum samples from negative individuals. Both peptides were able to detect infected individuals in all the stages tested, whether symptomatic or asymptomatic. Moreover, when comparing specificity with two different recombinant proteins, named rTc964 and rLm964, peptides showed a similar specificity value performance in relation to rTc964, where wasn’t observed reactivity with serum samples from active cutaneous leishmaniasis and cured cutaneous leishmaniasis.

Finally, Majeau et al. (2024) identified protein sequences present in the *T. cruzi* genome that are useful for diagnosis. Sequences evaluated correspond to 14 genomes from the main parasite’s lineages, and the selected proteins were ordered in peptide microarrays. Subsequently, the peptides were grouped into mixtures containing 6 to 22 peptides, and tested using an ELISA assay, compared with the commercial Chagastest ELISA test. A second ELISA was performed to evaluate the reactivity of the best peptide mixture. Serological panel included samples from different geographic regions, totalizing 64 positive samples and 51 negative samples. Mixtures 12 and 14 demonstrated the best results, with 65% sensitivity and 100% specificity, while Chagatest showed a 45% sensitivity and 100% specificity. In the second assay, peptide mixture 14 had 72.7% sensitivity and 87.5% specificity.

## Peptide-base Point-of-care (POCT) assays

Point-of-care (POCT) is a diagnostic strategy developed for rapid and accurate detection of diseases, capable of identifying the presence or absence of a particular antigen. (Goble and Rocafort 2017). Although this diagnostic tool has already been used for more than a decade for other diseases, such as HIV, syphilis, and hepatitis B and C, researchers have shown interest in expanding this technological innovation to include other diseases since the COVID-19 pandemic reinforced the importance of this assay (Brasil 2022; Nichols 2021). Two articles using peptides in this platform for CD diagnosis were found in the literature, in which the authors attempted to revolutionize their research by reconciling the optimization of clinical practice with the rapid delivery of a diagnosis.

A study by Bhattacharyya et al. (2018) was the first to test peptides in a POCT assay. In their work, the peptide TSSApep-II/V/VI, which had been previously tested by Bhattacharyya et al. (2014), was adapted into a new lateral flow immunochromatographic rapid diagnostic test (RDT) called Chagas Sero K-SeT RDT. To access serological reactivity, a panel composed of 336 serum samples from infected individuals and 58 serum samples from healthy individuals was used. Among the positive serum samples derived from paired maternal and cord blood tested, Chagas Sero K-SeT RDT detected 89 (89/131) infected individuals with 100% concordance between maternal and cord blood. Regarding serum samples derived from adult chronic infection, it detected 52.5% (21/40) of individuals without evidence of cardiomyopathy and 74.1% (60/81) of individuals with cardiomyopathy. Moreover, Chagas Sero K-SeT RDT showed reactivity with only 7 (7/65) serum samples among the Peruvian samples. Finally, this test showed a specificity \of 96.5%.

Continuing the above-cited studies with POCT, Murphy et al. (2019) evaluated the Chagas Sero K-SeT RDT using serum samples from the Chaco region of northern Argentina. A total of 393 positive samples were tested, in which the seroprevalence of Chagas Sero K‑SeT RDT was 69.5%. Moreover, Chagas Sero K-SeT RDT performed better when compared to TSSApep-based ELISA, given that Chagas Sero K-SeT RDT detected 61% of the positive cases, while only 34% of the cases were detected using TSSApep-based ELISA. Table 1 summarizes the main points of the above-cited studies.

**Table 1:** Peptide applied in the serodiagnosis of CD

Peptide name/Amino acid sequence	Origin protein	Obtaining method	Serological panel (positive/negative/cross-reactions serum samples)	Test used	Results	Author/Country
TcD synthetic/AEPKSAEPKPAEPKSGCG	TcD protein	Chemical Synthesis	9 T*. cruzi*-positive acute serum samples 120 T*. cruzi*-positive chronic serum samples 32 T*. cruzi*-negative serum samples Cross-reactive diseases: 10 malaria serum samples 16 mycobacterial serum samples 24 VL serum samples 15 CL serum samples	ELISA	Recognized by 96.7% of CD serum samples 3.3% of serum from non-Chagas individuals showed reactivity with the synthetic peptide	Burns et al. (1992)/United States
TcD/AEPKSAEPKP PEP2/GDKPSPFGOAAAGDKPSPFGQA	TcD and B13 proteins	Chemical Synthesis	179 T*. cruzi*-positive serum samples 81 T*. cruzi*-negative serum samples Cross-reactive diseases: 12 CL serum samples 11 VL serum samples 7 leprosy serum samples 7 tuberculosis serum samples	ELISA	TcD Sensitivity: 93.8% Specificity: 97% PEP2 Sensitivity: 91.6% Specificity: 98% Combination of TcD and PEP2 Sensitivity: 99.4% Specificity: 99%	Peralta et al. (1994)/Brazil
R-13/EEEEDDDMGFGLFD	Ribosomal P protein	Chemical Synthesis	7 T*. cruzi-*positive acute serum samples 7 T*. cruzi-*positive congenital serum samples 72 T*. cruzi-*positive chronic serum samples 75 T*. cruzi-*positive Bolivian serum samples 445 T*. cruzi*-negative serum samples	ELISA	IgM Acute: 7 Congenital: 0 Chronic: 14 IgG Acute: 7 Congenital: 7 Chronic: 72 86% of the congenital and 60% of the chronic serum samples recognized R-13 49% of serum samples from blood donors showed reactivity with R-13	Aznar et al. (1995)/France
2/D/E/GDKPSPFGQAAAGDKPSPFGQAGCGAEPKSAEPKPAEPKSGCGKAAIAPAKAAAAPAKAATAPA TcLo1.2/GTSEEGSRGGSSMPSGTSEEGSRGGSSMPA	TcD, L19E Ribosomal and B13 proteins	Chemical Synthesis	240 T*. cruzi*-positive serum samples 149 T*. cruzi*-negative serum samples Cross-reactive diseases: 8 CL serum samples 8 VL serum samples 8 malaria serum samples 8 tuberculosis serum samples	ELISA	2/D/E peptide Sensitivity: 99.6% Specificity: 99.33% TcL 1.2 has satisfactory results when exhibiting the reactions of the four epitopes. However, it exhibits reduced optical density and sensitivity values in relation to the tetrapeptide	Houghton et al. (1999)/United States
2/D/E-2/GDKPSPFGQAAAAGDKPSFGQAGCGAEPKSAEPPAPKSGKAAIAPAKAAAAPAKAATAPA	TcD and L19E Ribosomal proteins	Chemical Synthesis	9 T*. cruzi*-positive acute serum samples 31 T*. cruzi*-positive chronic serum samples 107 T*. cruzi*-negative serum samples Cross-reactive diseases: 7 rubella serum samples 7 chicken-pox serum samples 12 viral hepatitis serum samples 4 mumps serum samples 4 cytomegalovirus infection serum samples 12 AIDS serum samples 5 dengue serum samples 6 measles serum samples	ELISA	IgM The synthetic peptide was recognized by all serum samples from the acute infection IgG 12.9% of samples from chronically infected individuals showed reactivity with the 2/D/E peptide	Betonico et al. (1999)/Brazil
12,636/MATTLEEFSAKLDRL 12,637/DRLDAEFAKKMEEQNK 12,638/EQNKKFFADKPDESTL/ 12,639/ESTLSPEMKEHYEKFEK 12,640/KFEKMIQEHTDKFNKKM 12,641/NKKMHEHSEHFKAKFAE 12,642/KFAELLEQQKNAQFPGK	KMP11 protein	Chemical Synthesis	20 T*. cruzi*-positive chronic serum samples 10 T*. cruzi*-negative serum samples Cross-reactive diseases: 10 VL serum samples 5 tuberculosis serum samples 5 malaria serum samples	ELISA	Only peptides 12,638 and 12,642 were recognized by positive serum samples	Thomas et al. (2001)/Spain
R3/MRQLDTNVERRALGEIQNV S1/STPSTPADSSAHSTPSTPV	Cha protein and repeats of shed acute-phase antigen of *T. cruzi*	Chemical Synthesis	79 T*. cruzi*-positive chronic serum samples 10 T*. cruzi*-negative serum samples 6 T*. cruzi*-negative serum samples of individuals with idiopathic dilated cardiomyopathy Cross-reactive diseases: 10 VL serum samples	ELISA	There was low binding of anti-S1 antibodies in relation to anti-R3 in all serum samples R3 Sensitivity: 92.4% Specificity: 100%	Gironès et al. (2001)/Spain
p17 (R-COOH)/ - p17 (R-CONH2)/ - p18/ -	SAPA	Chemical Synthesis	20 T*. cruzi*-positive serum samples 20 T*. cruzi*-negative serum samples Cross-reactive diseases: 20 leishmaniasis serum samples	ELISA	p17 (R-COOH) Sensitivity: 100% Specificity: 100% p17 (R-CONH2) Sensitivity: 100% Specificity: 100% p18 Sensitivity: 100% Specificity: 100%	Hernández-Marin et al. (2003)/Cuba
P1/PSPFGQAAAGDK P2/AEPKPAEPKS	-	Chemical Synthesis	82 T*. cruzi*-positive serum samples 44 T*. cruzi*-negative serum samples Cross-reactive diseases: 20 toxoplasmosis serum samples 6 leprosy serum samples 15 HIV-1 serum samples 5 HIV-2 serum samples 20 HCV serum samples 20 HTLV-I serum samples	ELISA	P1 Recognized 69% of positive samples from Colombia (31/45) and 86% of positive samples from Brazil (32/37) P2 Recognized 49% of positive samples from Colombia (22/45) and 89% positive samples from Brazil (33/37)	Hernández-Marin et al. (2006)/Cuba
RP1/KKKLADRAFLDQKPEGVPLRELPLDDDSDFVAMEQERRQLLEKD PRRNAREIAALEESMNARAQELAR RP2/LIGTEAHMDSSSDSSAHSTPSTPADSSALSTPSTPADSSAHSTP STPADSSAHSTPSTPAGHGATGMVLILPD RP5/ADAQKSFNPSTDKLKINQQNKPHIANNKQKTTLEKTQTEQKTAP FGQAAAGDKPSLFGQA	H49, Ag1, FRA, JL7, SAPA, B13 and Ag2 proteins	Chemical Synthesis	32 T*. cruzi*-positive serum samples 32 T*. cruzi*-negative serum samples Cross-reactive diseases: 15 CL serum samples	ELISA	*T. cruzi* positive samples demonstrate reactivity for each synthetic antigen tested	Camussone et al. (2009)/Argentina
3972/FGQAAAGDKPPP 6303/FGQAAAGDKPAP 3973/FGQAAAGDKPSL 3963/FGQAAAGDKLSL 6173/FGQAAAGGKPSL	TcCA-2 protein	Simultaneous method of multiple peptides in solid phase	11 T*. cruzi*-positive acute serum samples 122 T*. cruzi*-positive chronic serum samples 50 T*. cruzi*-negative serum samples Cross-reactive diseases: 21 leishmaniasis serum samples 11 tuberculosis serum samples 11 malaria serum samples 15 serum samples of individuals with autoimmune disorders (Rheumatoid arthritis) 38 individuals with nonchagasic cardiac disorders serum samples	ELISA	The serum samples from acute phase dind’t recognized the peptides IgG: Peptide 3972 Sensitivity: > 90% Specificity: > 98% Peptide 6303 Sensitivity: > 90% Peptide 3973 Sensitivity: > 90% Peptide 3963 Sensitivity: 30% Peptide 6173 Sensitivity: 5%	Thomas et al. (2012)/Spain
P013/EDDDDDFGMGALF R13/EEEDDDMGFGLFD JL18/AYRKALPQEEEEDVGPRH JL19/VDPDFCRSTTQDAYRPVDP P0b/AESEE	TcP2b, TcP0 and JL9 proteins	Chemical Synthesis	228 T*. cruzi*-positive chronic serum samples 108 T*. cruzi*-negative serum samples Cross-reactive diseases: 5 VL serum samples 4 ML serum samples 19 serum samples from individuals with autoimmune diseases 16 cardiomyopathies of non-Chagas etiology serum samples 5 serum samples with another disease, such as juvenile diabetes, schistosomiasis, idiopathic megaesophagus, and South American blastomycosis	ELISA	P013 Sensitivity: 82.5% Specificity: 97.2% R13 Sensitivity: 61.4% Specificity: 85.1% JL18 Sensitivity: 37.3% Specificity: 78.7% JL19 Sensitivity: 40.4% Specificity: 75% P0b Sensitivity: 28.5% Specificity: 86.1%	Longhi et al. (2012)/Argentina
C6_30_cons/QRMSNASGGGGGG–MRQNE A6_30_col/–-ENSANPPPPDRSLPTP B2_30_y/FFQPQPQPQPQPQPQQF B9_30_cl/MDDDDD–ETYRGG	TSSA protein	Chemical Synthesis	66 T*. cruzi*-positive serum samples 24 T*. cruzi*-negative serum samples Cross-reactive diseases: 14 CL serum samples 14 VL serum samples	ELISA	A6_30_col Sensitivity: 100% Specificity: 91.9% C6_30_cons Sensitivity: 95.8% Specificity: 92.7% B2_30_y Sensitivity: 80% Specificity: 94% B9_30_cl Showed a low reactivity with both TcI and TcII	Mendes et al. (2013)/Brazil
CRA-1/AAKQKAAEAAAKQKAAEC CRA-2/AAKQRAAEAAAKQRAAEC FRA-1/ADRAFLDQKPERVPC	Cytoplasmic repetitive antigen (CRA) and flagellar repetitive antigen (FRA) proteins	Chemical Synthesis	31 T*. cruzi-*positive chronic serum samples 12 T*. cruzi*-negative serum samples Cross-reactive diseases: 14 CL serum samples	ELISA	CRA-1 Sensitivity: 100% Specificity: 100% CRA-2 Sensitivity: 100% Specificity: 100% FRA-1 Sensitivity: 91.6 Specificity: 60%	Bottino et al. (2013)/Brazil
TSSApep-I/GTDKKTAAGGTPSPSG TSSApep-II/V/VI/GTENKPATGEAPSQPG TSSApep-III/GTEKKAAAGEAPSPSG TSSApep-IV/GTDKKTAAGEAPSPSG TSSApep-V/VI/GTENKPAAGEAPSQPG	TSSA protein	Chemical Synthesis	186 T*. cruzi*-positive chronic serum samples 31 T*. cruzi*-negative serum samples	ELISA	4/20 (20%) sera from Ecuador reacted with TSSApep-II/V/VI 1/12 serum from Venezuela reacted with TSSApep-IV 1/34 serum from Colombia reacted with TSSApep-IV TSSApep-II/V/VI and TSSApep-V/VI were recognized by sera from Brazil, Ecuador, and Argentina	Bhattacharyya et al. (2014)/ United Kingdom
p36-50/ SGTENKPATGEAPSQ	TSSA protein	Chemical Synthesis	70 T*. cruzi*-positive chronic serum samples 38 T*. cruzi*-negative serum samples	ELISA	The peptide was recognized by most positive samples	Balouz et al. (2015)/Argentina
pc1/APFGQAAAGDKPSPF pc2/AAAPAKAAAAPAKAA pc3/EPKSAEPKPAEPKSA p6/TTNAPSRLREIDGSL p7/KLGKSVGLTAALSPR p13/DSAKGKATGSSAGED p24/AKPPAESPFKSVFGA	B13, Ag2, CA-2, PEP2, Ribo L19, TcD, Ag13, mucin TcMUCII, hypothetical conserved protein, trans-sialidase, hypothetical protein and n96 proteins	Chemical Synthesis	62 T*. cruzi-*positive chronic serum samples 16 T*. cruzi*-negative serum samples Cross-reactive diseases: 19 TL serum samples	ELISA	The results for these peptides ranged from 3.23 to 91.98% sensitivity and 93.8 to 100% specificity	Mucci et al. (2017)/Argentina
Peptide 1/AGKESKGEKEGENVSEAEKEGSHGNVDEEAAGKNGGN Peptide 2/KEPTDDEAKTKKRNEQKEAENANNTKEEPDEEEVK Peptide 3/ATDDEAKTKKRNEQKEAENANNTKEEPDEEEAKKA Mix II (peptides 2 and 3)	Mucin-associated surface proteins	Chemical Synthesis	53 T*. cruzi*-positive chronic serum samples 25 T*. cruzi*-negative serum samples Cross-reactive diseases: 22 TL serum samples 23 VL serum samples	ELISA	Peptide 1 Sensitivity: 60.38% Specificity:100% Peptide 2 Sensitivity: 100% Specificity: 97.14% Peptide 3 Sensitivity: 72.86% Specificity: 67.14% Mix II Sensitivity: 100% Specificity: 100%	Elisei et al. (2018)/Brazil
TSSA II/V/VI/GTENKPATGEAPSQPG	TSSA protein	Chemical Synthesis	131 T*. cruzi-*positive serum samples from paired mother and cord blood 121 T*. cruzi-*positive chronic serum samples 84 T*. cruzi-*positive serum samples from different countries 58 T*. cruzi*-negative serum samples	Rapid test	RDT results were positive in 68.7% (57/83) and 66.7% (32/48) of mothers 52.5% (21/40) of those without evidence of cardiomyopathy had positive RDT results compared with 74.1% (60/81) of those with cardiomyopathy There was no significant difference in the prevalence of positive RDT in samples from southern Peru 4/49 (8.2%) and northem Peru 3/16 (18.8%) 2/58 serum samples were positive and displayed a specificity of 96.5%	Bhattacharyya et al. (2018)/ United Kingdom
TSSApep-II/V/VI/GTENKPATGEAPSQPG	TSSA protein	Chemical Synthesis	393 T*. cruzi-*positive serum samples from	Rapid test	69.5% seroprevalence (273/393)	Murphy et al. (2019)/United Kingdom
TcNV/ - TcKP/ -	Tc964 protein	Chemical Synthesis	63 T*. cruzi-*positive chronic serum samples 6 T*. cruzi*-negative serum samples Cross-reactive diseases: 12 ACL serum samples 11 CCL serum samples	ELISA	The peptides were recognized by all positive serum samples	Ruiz-Márvez et al. (2020)/Colombia
Mix 1/ - Mix 2/ - Mix 3/ - Mix 4/ - Mix 9/ - Mix 12/ - Mix 14/ -	CA-2/B13, SAPA, Antigen 1, TSSA, Ribosomal P2, Ribosomal L19 and JL8	Chemical Synthesis	64 T*. cruzi-*positive chronic serum samples 51 T*. cruzi*-negative serum samples	ELISA	Mix 1 Sensitivity: 30% Specificity: 90.9% Mix 2 Sensitivity: 60% Specificity: 81.8% Mix 3 Sensitivity: 65% Specificity: 100% Mix 4 Sensitivity: 60% Specificity: 100% Mix 9 Sensitivity: 60% Specificity: 100% Mix 12 Sensitivity: 65% Specificity: 100% Mix 14 Sensitivity: 65% and 72.7% Specificity:100% and 87.5%	Majeau et al. (2024)/United States

*ACL* active cutaneous leishmaniasis, *CCL* cured cutaneous leishmaniasis, *CL* cutaneous leishmaniasis, *VL* visceral leishmaniasis, *TL* tegumentary leishmaniasis, *ML* mucocutaneous leishmaniasis, *HIV-1* human immunodeficiency virus type 1, *HIV-2* human immunodeficiency virus type 2, *HCV* hepatitis C virus, *HTLV-1* human T-cell leukemia virus type I, *RDT* rapid diagnostic test

## Discussion

Although a number of CD diagnostic tools have been developed, most endemic regions are still affected by the underdiagnosis of *T. cruzi* infection (Suárez et al. 2022). In fact, endemic and non-endemic countries find it difficult to incorporate new technologies and have to rely on inefficient strategies, mainly due to monetary restrictions (Abras et al. 2022). Considering the different specifications approved in the current preparation of diagnostic kits, peptide-based antigens represent a viable alternative (Bhattacharyya et al. 2018; Murphy et al. 2019). The use of peptides for diagnostics offers some advantages over the use of recombinant proteins, given that recombinant proteins may trigger more false-positive results due to the larger size, in addition of having the possibility of misfolding and poor conformation of the protein, which can impact negatively in diagnostics tests (Pandey et al. 2021). Moreover, a standardized diagnosis could be more easily achieved using peptides considering batch variation is more unusual when working with peptides as compared to recombinant proteins. Furthermore, recombinant proteins usually require a living organism as an expression system, contrasting with methods of obtaining peptides that normally do not require living organisms for their production (Francis and Page 2010). As such, the identification and validation of short peptides as possible antigenic targets in the serological diagnosis of CD has attracted the attention of many scientists.

Researchers have made efforts to screen promising new peptides for CD diagnosis. The data presented in the studies described above indicate that many peptides tested were promising for the CD diagnosis, as they indicated sensitivity and specificity greater than 90%. It is known that antigens from totally inactive parasites can cross-react with antigens from other protozoans, such as *Leishmania* spp., which is one of the main causes of cross-reaction in serological tests (Schijman et al. 2022). Studies, such as those developed by Aznar et al. (1995), Betonico et al. (1999), Hernández-Marin et al. (2003) and Elisei et al. (2018), demonstrated that the use of peptides can reduce the occurrence of cross-reactivity, maintaining the percentage of reliable positive and negative results. Although many results were favorable, it cannot yet be inferred which of these peptides has the best diagnostic performance, due to several reasons, such as the different sample panels used, different concentrations of peptides and different diagnostic protocols.

Among the studies described above, the majority used a serological panel composed of serum from individuals in the chronic phase, detecting the presence of IgG class antibodies. In fact, only four studies included serum samples from individuals in both the acute and chronic phase, detecting the presence of IgM and IgG, respectively.

**Fig. 1:**
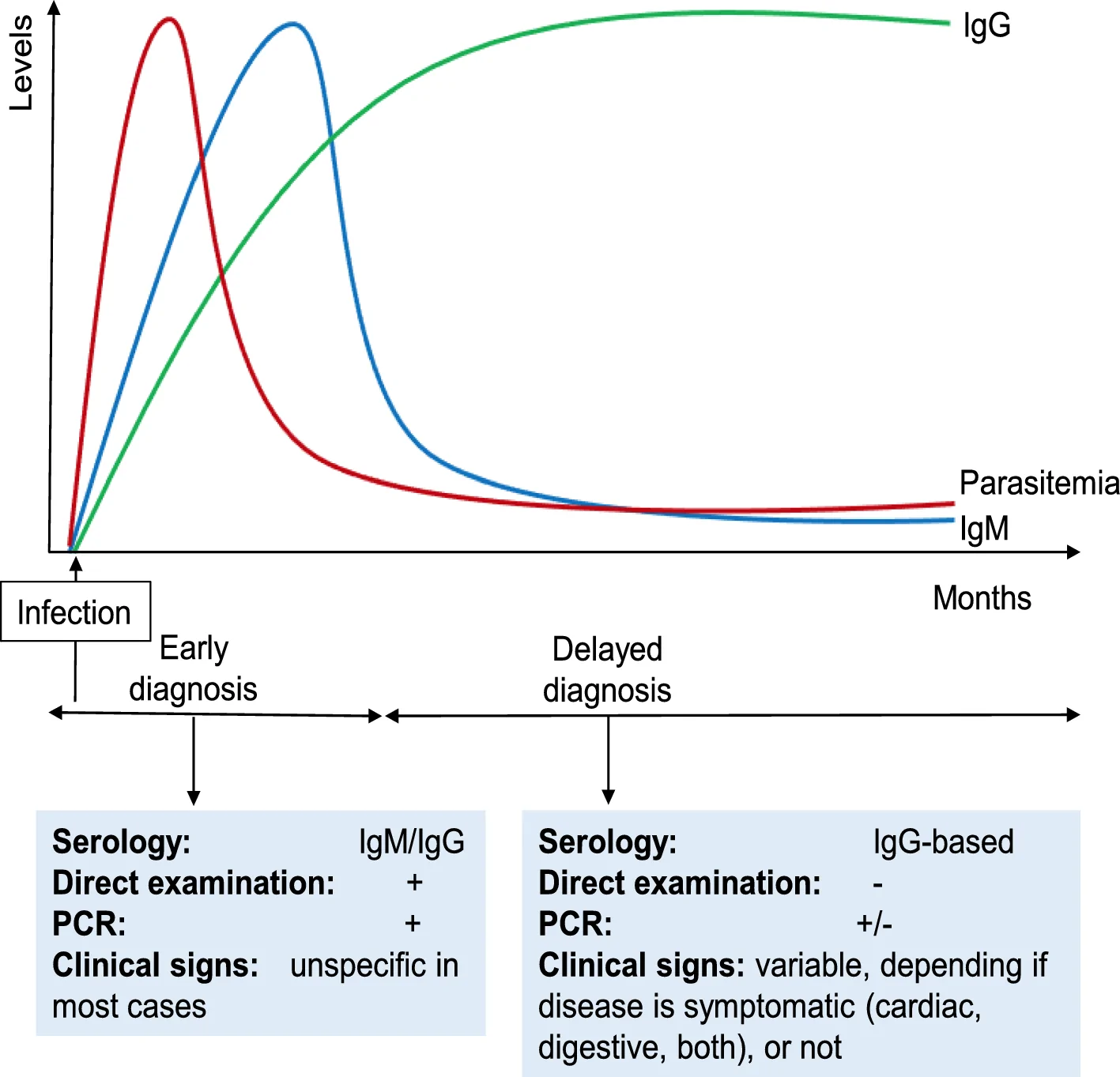
Dynamics of parasitemia and sera antibodies, which are the basis for detecting the parasite or its DNA, as well as antibodies, during acute and chronic *Trypanosoma cruzi* infection. During acute infection, parasitemia is high, allowing for direct examination- and PCR-based diagnosis. Antibody-based methods detecting mostly IgM are also used. Acute phase is the best window for diagnosis for increased treatment efficacy. At the chronic stage, low grade parasitemia does not allow for direct examination, and PCR-based methods are variable in sensitivity. Antibody-based specific IgG serology is widely used. All testes are laboratory-based, with no point-of-care tests currently available. No tests for disease prognosis are currently available

Therefore, more studies are still needed to investigate the diagnostic performance of the different peptides, using characterized serum samples from both the acute and chronic phase. Based on the dynamics of parasitemia and sera antibodies (Fig. 1), the reality of serological tests for Chagas currently shows that, in addition to the need to perform more than one test to diagnose chronic infection, there is still a major bottleneck in diagnosing the disease in the acute phase. The lack of an accurate serological diagnosis in the acute phase of CD highlights a major problem, since the identification of *T. cruzi* through direct and indirect parasitological methods is more laborious, in addition to the social vulnerability of some individuals making it impossible to diagnose. In this sense, it is clear that it is still necessary the development of a single biomarker capable of diagnose the infected individual in both phases of the disease.

It should be noted that many reasons can interfere with the sensitivities of the tests, mainly in relation to the selection of antigens with better immunoreactivity, and the ability to generate less interference in the tests (Ferreira et al. 1991), where the similarity of epitopes that exist between diferente species, such as *Leishmania spp* and *T. cruzi* (Granjon et al. 2016; Ferreira et al. 2001), can negatively impact in diagnostic accuracy. In this sense, the search for epitopes capable of presenting high sensitivity and specificity is extremely importante. In that regard, it is possible to observe that most of the above-mentioned peptides come only from commonly used proteins, such as MAP, SAPA, CRA, FRA, TSSA, B13 and TcD, of which many of them composes commercial kits for the disease, such as Chagas ELISA IgG + IgM® (Vircell®, Granada, Spain) and IgG-ELISA® (NovaTec Immunodiagnostica GmbH; Dietzenbach, Germany). In this sense, since many peptides are derived from proteins already widely used, their excellent diagnostic performance was already expected. However, further studies using the same serological panel are needed to evaluate the performance of the peptides and their source proteins. Moreover, it is known that the amino acid sequence of MAP and FRA, for example, presents 40% and 70% similarity with *L. infantum*, as well as 52% and 63% similarity with *L. braziliensis*, respectively (Hernández et al. 2010). This similarity between species can be a possible explanation for some unsatisfactory results presented using some peptides, such as FRA-1 and 6173 peptides. especially regarding specificity values.

It is evident that most peptides have different amino acid sequences, except TcD, P1, P2, pc3, RP5 and 3973, which share the same sequences or present some small variation. This reflects the effectiveness of these peptides, since these sequences resulted in high sensitivity and specificity values, even with different serological panels employed in serological tests, confirming their potential as an antigenic marker. Furthermore, peptides that show good results motivated the development of commercial tests, such as BioElisa Chagas (Biokit, Barcelona, Spain), which uses recombinant antigen composed of TcD, TcE, PEP2 and TCLi1-2, achieving 98% sensitivity and 100% specificity (Duarte et al. 2014; Houghton et al. 1999), and Chagas Sero K-SeT, which employed the peptide TSSApep-II/V/VI (Murphy et al. 2021).

When developing diagnostic tests, the genetic variability of *T. cruzi* must be taken into account, a factor that interferes with the accuracy of the results, highlighting the importance of studies that evaluate the antigenic structures of this parasite (Mendes et al. 2013). The broad parasite genetic lineage in different geographic areas can directly interfere with the diagnostic performance, which may negatively impact the sensitivity of the tests. Despite that, most serological tests developed to date does not focus on the use of markers that are genetically conserved among the most diverse strains. In fact, biomarkers originating from highly conserved proteins can provide excellent results, being promising candidates for the development of a universal diagnosis (Majeau et al. 2024). Indeed, the investigation of conserved peptide portions is essential for the incorporation of antigens able to be recognized by antibodies produced against different parasite strains (Rodríguez-Bejarano et al. 2021; Reis-Cunha et al. 2014; Mendes et al. 2013). The genetic variability of each tested population must also be taken into account since lifestyle, nutritional and immunological status, and previous disease history, are important factors for the progression of the disease in the infected individual, which can impact directly in the diagnosis (Magalhães et al. 2022). In fact, the study developed by Bhattacharyya et al. (2018) showed that positive serum samples from different geographical areas recognized differently the peptides under study, impacting the diagnostic performance of each. This reinforces the need to expand the study population in future studies.

In addition, a more accurate prediction and selection of epitopes to be incorporate in diagnostic kits is of great relevance, as it can enable the manufacture of more specific reagents, capable of discriminating the clinical phase of the disease (Balouz et al. 2017). The characterization of immunodominant epitopes contributes to the epidemiological search for each genetic lineage of the parasite, favoring the development of control and prevention strategies, as well as the production of more sensitive diagnoses (Suárez et al. 2022). In fact, studies that provide a more in-depth characterization of epitopes, as well as broad testing in different populations, are essential for better guidance in the development of new biomarkers for diagnosis (Ricci et al. 2023).

Diagnostic techniques can exhibit inaccessible conditions, especially in remote areas where laboratories are precarious, with few resources, and the lack of trained professionals. Therefore, rapid diagnostic test development would overcome this hindrance, considering this assay does not require equipment to be set up to carry it out, thus reducing operating costs and providing faster results. Health agents could diagnose individuals in remote locations, enabling a better prognosis of the infection. There are still many limitations in obtaining an accurate CD serological diagnosis, suggesting some measures could be implemented, such as manufacturing low-cost reagents, obtaining new biomarkers, and developing rapid tests. Furthermore, improved ELISA techniques would allow more accurate diagnoses to be made, reducing the number of tests and giving more people access to an early and accurate diagnosis. Having better access to an accurate CD diagnosis could help control the disease.

This review brings together information on the use of peptides in serological diagnosis in infected individuals. Most studies were efficient and demonstrated promising peptides capable of providing an optimized, high-throughput differential diagnosis. Therefore, the use of peptides in serological tests has been increasingly used to improve *T. cruzi* identification and disease monitoring.

## Data Availability

Not applicable.
